# Global expert consensus on the importance of secondary stroke prevention: challenges, care coordination and unmet needs for non-cardioembolic ischaemic stroke survivors

**DOI:** 10.1093/esj/aakaf012

**Published:** 2026-01-01

**Authors:** Valeria Caso, Julio Agredano, Charlotte Cordonnier, Jesse Dawson, Gian Marco De Marchis, Karl Georg Haeusler, Teruyuki Hirano, Liping Liu, Jaime Masjuan, Nenad Nikolić, Hariklia Proios, Adam Siger, Marianne Helén Tangen, Arlene Wilkie

**Affiliations:** Neurology-Stroke Unit, Saronno Hospital, ASST Valle Olona, Piazzale Borella, 1, Saronno 21047, Italy; Fundación Freno al ICTUS, Av Soller 97, Madrid 28290, Spain; Hopital Salengro, Lille University Hospital, Bd Emile Laine, Lille 59000, France; School of Cardiovascular and Metabolic Health, College of Medical, Veterinary & Life Sciences, Queen Elizabeth University Hospital, University of Glasgow, Glasgow G51 4TF, United Kingdom; University Research and Teaching Hospital, Clinic for Neurology, HOCH-Kantonsspital St. Gallen, Rorschacherstrasse 95, St. Gallen 9010, Switzerland; Department of Neurology, University of Ulm, Ulm 89081, Germany; Department of Stroke and Cerebrovascular Medicine, Kyorin University School of Medicine, 6-20-2 Shinkawa, Mitaka, Tokyo 181-8611, Japan; Department of Neurology, Beijing Tiantan Hospital, Capital Medical University, 119 West Road, 4th South Ring, Fengtai District, Beijing 100070, China; Neurology Department, Hospital Universitario Ramón y Cajal, Carretera de Colmenar, KM 9.1, Madrid 28220, Spain; Department of Neurology EEG Cabinet, General Hospital Ćuprija, Miodraga Novakovića 78, Ćuprija 35230, Serbia; Stroke Alliance for Europe, Rue Washington 40, Brussels 1050, Belgium; Fundacja Udaru Mózgu, Milionowa 14, Lodz 93-113, Poland; Raakhaugen omsorgssenter, Molde Kommune, Raadhusplassen 1, Molde 6413, Norway; Stroke Alliance for Europe, Rue Washington 40, Brussels 1050, Belgium

**Keywords:** secondary stroke prevention, recurrent stroke, ischaemic stroke, stroke prevention, unmet need, modified Delphi, expert consensus

## Abstract

**Introduction:**

Despite advances in clinical care and treatment options, recurrent stroke risk remains significant. The unmet needs and challenges in secondary stroke prevention (SSP) after a non-cardioembolic ischaemic stroke are not fully understood, leaving many patients at risk of stroke recurrence. This study summarises expert consensus on the challenges in current SSP treatment and management.

**Patients and methods:**

We conducted a 2-round modified Delphi study with 13 international stroke experts. This multidisciplinary panel included stroke neurologists, a stroke nurse, a dementia care nurse with lived experience and patient advocacy group representatives. The Delphi co-chairs developed 11 statements which were presented to the experts. Agreement was sought through a 2-round, anonymous survey and a final consensus discussion.

**Results:**

All 11 statements achieved consensus after the 2 survey rounds. The statements addressed key areas including the burden of recurrent stroke, treatment and lifestyle interventions, management of stroke care and future needs to enhance SSP.

**Conclusion:**

This is the first Delphi-based global consensus focused specifically on unmet needs in SSP. The experts agreed on several challenges—notably, recurrent stroke risks—and consistently emphasised that the impact of recurrent stroke is underappreciated. This Delphi panel’s strong consensus underscores the real-world barriers, clinical inefficiencies and unmet needs that remain in SSP treatment and management. Addressing these challenges will require sustained investment in SSP treatments, education and innovation.

## Graphical Abstract

**Graphical abstract:**
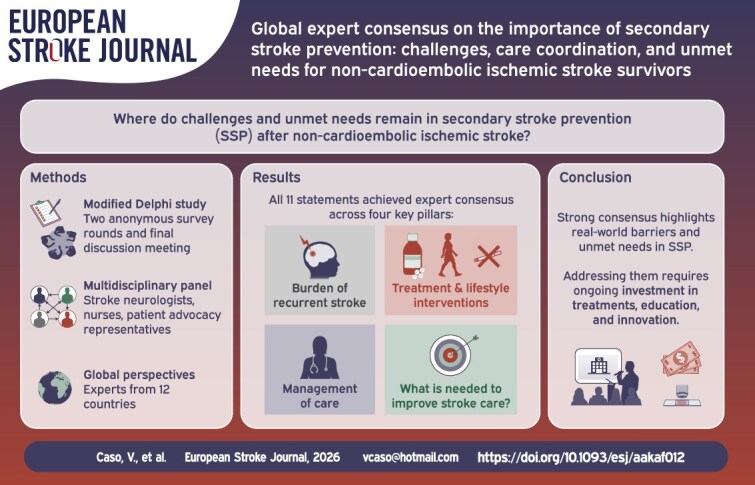

## Introduction

Globally, stroke remains the second-leading cause of death, and the third-leading cause of death and disability combined.^1^ Ischaemic strokes are caused by a blood vessel obstruction resulting in reduced or interrupted blood flow to a region of the brain. These account for approximately 65% of all incident stroke cases globally, and the majority of these strokes are non-cardioembolic in origin—indicating that a blood clot forms outside the heart.^2–4^

Individuals who have experienced a stroke have a significant risk of stroke recurrence. To reduce this risk, antiplatelet therapy is used as a preventive measure alongside risk factor management as the current standard of care (SoC).^5–7^ However, even with current treatments and advances in clinical care, the risk of recurrent stroke remains substantial, as about 1 in 10 stroke survivors experience another ischaemic stroke within the first year.^8^ By 2050, the number of stroke incident cases is expected to increase by 81% compared to 2021 levels, primarily due to the aging population and increasing stroke survivorship. Consequently, the burden of secondary stroke is expected to intensify; recurrence rates are anticipated to rise accordingly.^9^

Stroke management includes stroke survivors alongside several other stakeholders (e.g., neurologists, stroke nurses, primary care physicians, patient advocacy groups, caregivers and allied care professionals). However, there remains a significant lack of clarity and shared understanding among these groups regarding the challenges of secondary stroke prevention (SSP), leading to fragmented approaches and suboptimal patient outcomes. It is essential to increase understanding of the unmet needs and challenges in SSP following a non-cardioembolic ischaemic stroke, particularly in relation to current SoC.

This study represents the first comprehensive effort to capture global expert consensus on the unmet needs in SSP. A global panel of stroke neurologists, a stroke nurse, a dementia care nurse and patient group representatives was assembled to discuss the unmet needs and challenges of SSP. The panel also included lived experience, with one expert being a stroke survivor and another serving as a caregiver for a stroke survivor. A Delphi study was undertaken to gain consensus on specific aspects of non-cardioembolic ischaemic stroke recurrence, including the real-world barriers, clinical inefficiencies, care coordination gaps and associated clinical and economic burden.

## Methods

We designed a Delphi study to achieve consensus on the challenges in current SSP treatment and management. The Delphi method is a well-accepted approach of developing consensus across a panel of experts using multiple survey rounds and controlled feedback.^10^ This method was chosen to support structured discussion and explore areas of agreement among experts through a series of iterative rounds.

### Expert panel selection criteria and composition

We convened a multidisciplinary panel of 13 global experts: 8 stroke neurologists, 3 representatives from stroke patient advocacy groups, 1 stroke nurse and 1 dementia care nurse. Experts were selected based on their experience with stroke patients, geographic diversity and ability to provide in-depth insights into real-world SSP management pathways; 2 also contributed valuable perspectives as a stroke survivor and caregiver. Participants represented a broad range of countries: China, France, Germany, Greece, Italy, Japan, Norway, Poland, Serbia, Spain, Switzerland and the United Kingdom.

### Definition of consensus

A systematic review of a random sample of 100 Delphi studies found that a 75% agreement threshold was the median for defining consensus.^11^ Accordingly, consensus in this study was defined as at least 75% of experts (ie, 10 or more) agreeing with both the statement and its supporting evidence.

### Delphi study design and consensus process

Two Delphi panel co-chairs developed 11 starting statements relating to the unmet need and challenges associated with SSP, each supported by relevant evidence. Expert consensus was sought through a 2-round anonymous Delphi survey followed by a final consensus discussion meeting. All panel members participated in both Delphi survey rounds and the final consensus meeting, resulting in a 100% participation rate.

During the first survey round, experts were asked to review each statement by indicating whether they agreed with it and whether they agreed that the supporting evidence was robust. If they disagreed with either the statement or evidence, they were asked to explain why. All experts, regardless of agreement, were also invited to suggest improvements to the statement wording and evidence, highlight missing elements and provide any additional commentary they felt was needed.

Following round 1, expert feedback was collected and summarised. In round 2, statements that had not reached consensus were revised based on the expert feedback and recirculated. Expert responses were once again gathered and summarised. Once both survey rounds were complete, all original consensus statements were further refined based on comprehensive expert feedback and a refined version of each statement was developed. We then held a half-day virtual meeting, during which panel members reviewed, voted on and finalised the refined statements in an open, non-anonymous setting.

## Results—perspectives of the panel

### Consensus summary

The panel reached consensus on all 11 statements highlighting the unmet needs and challenges associated with SSP. Ten of the 11 statements achieved consensus (10 or more experts agreeing with both statement and evidence) during round 1 and were not recirculated. The remaining statement was revised based on expert feedback and re-circulated anonymously in round 2, at which point full consensus was achieved on the final statement.

During the half-day virtual meeting, all the statements that were refined prior to the virtual meeting demonstrated higher levels of expert agreement, both in terms of content and supporting evidence, compared to their original versions. The trend was consistent across all 11 statements. Therefore, all refined versions were adopted. Full expert agreement (100%) was achieved for all refined statements except statement 3, where 1 panellist disagreed with the supporting evidence.

### Burden of recurrent stroke

Statement 1: *Risk of recurrent stroke remains high in both the short and long terms. The recurrence rate has not substantially changed for decades despite advancements in acute stroke treatment, SSP and stroke care technologies.*

The risk of recurrent stroke remains high in both the short and long terms, with approximately 1 in 10 ischaemic stroke survivors having a second stroke within the first year, and approximately 1 in 5 having a second stroke within 5 years.^12–15^ Despite advancements in acute ischaemic stroke treatment and an increase in the use of secondary prevention medication, the stroke recurrence rate has remained almost unchanged for the last 20 years.^16^^,^^17^

Independent risk factors for stroke recurrence include smoking, hypertension, diabetes mellitus, a history of prior cerebrovascular events, higher stroke severity, hyperlipidaemia, chronic kidney disease, active cancer and air pollution.^16^^,^^18–21^

The expert panel emphasised that this statement is particularly relevant in certain high-risk subgroups, such as individuals with severe intracranial atherosclerosis and those at high risk of transient ischaemic attack (TIA).

Statement 2: *Stroke, including stroke recurrence, is one of the leading causes of long-term disability in adults worldwide.*

In 2020, stroke was the third-leading cause of death and disability worldwide, with almost 30% of all strokes being recurrent events.^22^^,^^23^ Globally, up to 50% of stroke survivors become chronically disabled, and nearly two-thirds of stroke survivors are discharged from the hospital with a disability.^24^^,^^25^

Overall disease burden remains high. The global disability-adjusted life years for ischaemic stroke were 70,357,911 in 2021.^26^ In addition, stroke survivors showed unfavourable results in patient-reported outcomes at 3 months after stroke, including anxiety, depression and global physical and mental health measures.^27^

Although stroke is already a leading cause of disability among adults, the experts stressed that its incidence is continuing to rise as the population ages. As a result, the burden of recurrent stroke is expected to grow, with recurrence rates projected to increase accordingly. This trend will have significant long-term implications for healthcare systems, stroke survivors and their caregivers.

Statement 3: *The impact of recurrent strokes on patients and caregivers is higher than for a first stroke, with an increased risk of death and mental health challenges.*

It has been shown that the mortality rate for patients increases following a recurrent stroke.^16^ The risk of death in the 30 days following a recurrent stroke is notably high, ranging from 23% to 41%.^28^

Recurrent ischaemic strokes often result in greater disability and have poorer outcomes than the first stroke, complicating recovery and rehabilitation.^22^ In addition, psychological factors such as post-stroke fatigue, depression, anxiety and fear of recurrent stroke can significantly impact the quality of life of stroke survivors and their caregivers.^29^^,^^30^

Statement 4: *The financial impact of recurrent strokes is high for both individuals affected by the stroke and the healthcare and social system, leading to high:*
 

- *direct costs (such as medical and support services)*,
- *indirect costs (such as loss of work for both patients and carers)*.

Compared to patients with a first ischaemic stroke, those with a recurrent ischaemic stroke had higher in-hospital and follow-up disability costs.^23^ Converted to 2021 United States dollars (USD), the mean in-hospital cost for each recurrent stroke patient was $23,145 USD, and the 1-year disability cost was $46,826 USD.^23^ These elevated costs are likely driven by the fact that the average length of stay for acute ischaemic stroke-related readmissions was longer than that of the initial hospitalisation.^31^^,^^32^

The experts highlighted that integrating healthcare systems with stroke support networks, such as patient support organisations, is essential not only for improving stroke patient recovery but also for delivering significant economic benefits. Effective care coordination reduces costly hospital readmissions, shortens rehabilitation time and minimises long-term dependency on healthcare services. By connecting patients with stroke support networks, healthcare systems can promote independence, ultimately lowering the financial burden on both healthcare providers and public health budgets.

### Treatment and lifestyle interventions

Statement 5: *Understanding the underlying cause of an individual’s stroke is crucial to ensure that appropriate secondary prevention interventions are prescribed.*

Determining the specific causal mechanisms of a patient’s stroke is essential for tailoring therapies that are effective at reducing recurrence and addressing individual risk factors like hypertension, diabetes and dyslipidaemia.^33^ In the coming years, treatment is likely to become more specific to the underlying stroke aetiology, and guidelines may need to be refined.^5^

Statement 6: *Lifestyle changes, tailored stroke support and continuous medication are key to preventing recurrent strokes. Ongoing education is necessary to further strengthen the engagement between patients and physicians, and to ensure adherence to the latest clinical guidelines.*

Committing to lifestyle modifications is an effective stroke prevention measure, with up to 87% of stroke risk being attributable to modifiable risk factors.^34^^,^^35^ While managing risk factors lowers mortality risk, long-term lifestyle changes can be difficult to implement and sustain with certain post-stroke effects. Broader stroke support and medication is important for ongoing prevention.^28^ Guidelines recommend that individuals who have experienced a stroke (without atrial fibrillation) should be prescribed antiplatelets and lipid-lowering treatments for long-term stroke prevention.^5^^,^^36^

Statement 7: *Implementing evidence-based SSP strategies can also benefit heart and brain health by lowering the risk of* *:*
 

- *neurological conditions like post-stroke cognitive decline and vascular dementia*,
- *cardiovascular conditions like myocardial infarction and peripheral artery disease*.

Protective effects against cognitive impairment have been observed in patients on the combination of antihypertensives, antithrombotic agents and lipid-lowering drugs.^37^ These same medications, when used for SSP, can also lower the risk of subsequent vascular events by 20–30%.^38–40^ It has been estimated that changes to diet and exercise following a stroke, along with optimised pharmacotherapy, could result in an 80% reduction in recurrent vascular events at 5 years.^41^

Statement 8: *As the antiplatelet SoC is associated with an increased risk of bleeding, the benefit/risk ratio must be considered when prescribing antiplatelet therapies for SSP.*

While dual antiplatelet therapy (DAPT) is considered the current SoC in some countries for short-term prevention of non-cardioembolic stroke recurrence, it is associated with a high early risk of major bleeding—particularly gastrointestinal bleeding.^42^ Furthermore, long-term use of DAPT (>90 days) has demonstrated no benefit over SAPT for recurrent stroke prevention and may increase the risk of major bleeding.^5^^,^^6^^,^^43^ However, SAPT is also associated with an increased risk of bleeding, with both all-site and gastrointestinal bleeding occurring significantly more frequently in patients receiving low-dose aspirin compared to those on placebo.^44^

While SSP involves many factors beyond medication, the experts agreed it was appropriate for this statement to focus on the increased bleeding risk associated with antiplatelet therapy. As the current SoC for SSP, and the most widely used treatment alongside antihypertensives, antiplatelets carry some of the most serious complications, particularly bleeding. When prescribing such treatments to stroke patients, the need for an extensive benefit–risk assessment was stressed, particularly in older or comorbid populations. This reinforces the urgent need for more precise risk-assessment tools and personalised treatment plans to reduce stroke recurrence and improve long-term patient outcomes.

### Management of care

Statement 9: *Optimal prevention of recurrent strokes requires healthcare professionals, stroke survivors and their carers to be “partners in prevention.” Regular patient reviews have been shown to support implementation of updated guidelines and improve patient outcomes.*

Implementing long-term secondary prevention guidelines has been shown to reduce mortality and recurrent cardiovascular events in individuals with non-cardioembolic ischaemic stroke or TIA.^45^ In addition, an intensified secondary prevention programme, including motivational interviewing, improved secondary prevention outcomes in patients with non-disabling stroke or TIA.^46^

Alongside regular patient reviews, the experts emphasised the value of post-stroke tools for ongoing patient evaluation that could improve outcomes. As an example, tools such as post-stroke checklists were highlighted, which are designed for completion by stroke survivors, with caregiver assistance if needed. Its purpose is to identify long-term challenges in stroke survivors and supports HCPs in facilitating timely and appropriate referrals for treatment.

Statement 10: *Access to the local care system and support should be clearly defined and coordinated across settings, to* *:*
 

- *maintain long-term engagement with healthcare professionals*,
- *improve adherence to current guidelines*,
- *ensure the supply of essential treatments and aids*.

To prevent recurrent strokes, patients must be supported as they navigate multiple healthcare settings, including the emergency department, stroke ward, rehabilitation centre, outpatient care and primary care.^47^ Lack of coordinated follow-up has been flagged as a barrier to accessing appropriate, ongoing stroke treatment and support. Improving patient follow-up will be essential for optimising SSP.^48^^,^^49^

Recent evidence suggests that comprehensive pragmatic care pathways, coupled with ongoing post-hospital involvement of the multidisciplinary stroke team, could reduce the longer-term health burden of stroke.^50^ For example, in the STROKE-CARD study, in addition to SoC, patients and their caregivers attended an outpatient appointment 3 months after the index event. This comprehensive session was conducted by a multidisciplinary team that included stroke physicians, nurses, physiotherapists, occupational therapists and speech therapists. This care programme reduced recurrent vascular events and improved QoL as well as functional outcomes at 12 months.^51^

### What is needed?

Statement 11: *Sustained investment in SSP management, support and new treatment options are needed to improve quality of life for patients and carers and achieve long-term cost savings. National stroke plans that include SSP, supported by clear guidelines and policies, will be required to support this goal.*

Experts emphasised that SSP should be individualised, as patient profiles can vary significantly. They also noted that emerging technologies such as artificial intelligence may play a valuable role in supporting these goals by helping detect strokes, assess severity and predict outcomes.^52^

## Discussion

This is the first Delphi-based global consensus explicitly focused on unmet needs in SSP. This Delphi study brought together a panel of experts, each of whom sees a different aspect of SSP burden in a different setting or country. The high level of consensus among the 13 experts underscores the widely recognised gaps in current SSP treatment and management, highlighting significant opportunities to enhance the quality of care. While agreement was reached on many major issues—including recurrent stroke risk and the limitations of current treatment approaches—experts consistently emphasised that the impact of recurrent stroke is often underappreciated, highlighting the need for increased focus on education and innovation in this area.

The modified Delphi method ensured that no single voice dominated, while ensuring every voice was heard. Similarly, anonymity was used to minimise dominance and group conformity during the survey rounds.^53^ While the Delphi panel participant pool was small, we endeavoured to ensure that it was representative of a wide range of differing global perspectives. Despite these strengths, the Delphi approach is not without important limitations. The iterative survey design, while useful for building consensus, may unintentionally narrow the diversity of viewpoints as minority perspectives can become diluted through repeated rounds of feedback. It is also recognised that individual contributions have the potential to be shaped by individual biases and the expert panel being composed primarily of stroke neurologists, may possibly introduce a disciplinary bias.

While this study was supported by Bayer, independence of the panel was protected by ensuring that all communications about the statements and facilitation of discussions between the panel were handled by an outside agency (Wickenstones Ltd.).

While generating this consensus statement, the experts easily agreed on the urgency of addressing recurrent stroke, which they all recognised as a significant unmet need from their respective viewpoints. Instead, the challenge was in finding robust data, which captures the lived experiences of individuals who experience recurrent strokes and the impact of SSP. There is a particular need for further investigation into the burden of illness related to recurrent stroke, as well as its economic impact and healthcare resource utilisation.

Ischaemic stroke rates remain high in contemporary settings.^54^^,^^55^ This, along with an aging global population and the growing burden of recurrent stroke on healthcare systems, suggests a pressing need to develop and implement new treatment options aimed at improving outcomes for both patients and caregivers.

These consensus statements can serve as a practical framework for delineating the current challenges and inefficiencies in SSP treatment and management. The expert panel emphasised the urgent need for healthcare stakeholders and policymakers to recognise the pivotal role of SSP and to prioritise it in healthcare planning and resource allocation.

## Conclusion

The expert panel’s strong consensus underscores a clear and urgent message: critical gaps remain in the treatment and management of SSP. To close these gaps, sustained investment in SSP treatments, support and innovation is essential—not only to improve outcomes and quality of life for stroke survivors and caregivers but also to deliver long-term cost savings to society and healthcare systems. Achieving this will require national stroke plans that prioritise SSP, backed by clear guidelines, policies and a commitment to action by all stakeholders involved in SSP.

The Delphi panel discussion conveyed a shared recognition that addressing these challenges and achieving meaningful progress in research, funding, policy and patient engagement will require collective commitment and collaboration across all sectors.

## Data Availability

No new data were created or analysed in this study. Data sharing is not applicable.
